# *Pneumocystis jirovecii* DNA in Serum for the Diagnosis of *Pneumocystis* Pneumonia in Patients with Hematologic Malignancies—A Retrospective Case Control Study

**DOI:** 10.3390/jof12070534

**Published:** 2026-07-20

**Authors:** Caroline Ström Turesson, Anna Grankvist, Helena Hammarström

**Affiliations:** 1 Department of Infectious Diseases, Institute of Biomedicine, Sahlgrenska Academy, University of Gothenburg, 405 30 Gothenburg, Sweden; caroline.s.strom@vgregion.se (C.S.T.); anna.grankvist@vgregion.se (A.G.); 2 Department of Clinical Microbiology, Sahlgrenska University Hospital, Region Västra Götaland, 402 34 Gothenburg, Sweden; 3 Department of Infectious Diseases, Sahlgrenska University Hospital, Region Västra Götaland, 416 85 Gothenburg, Sweden

**Keywords:** *Pneumocystis jirovecii* pneumonia, non-invasive diagnosis, serum polymerase chain reaction, β-D-glucan, hematologic malignancy

## Abstract

Diagnosis of *Pneumocystis jirovecii* pneumonia (PCP) relies on lower respiratory tract sampling, often requiring bronchoscopy. Given its invasive nature, non-invasive diagnostic methods are desirable. In this retrospective case–control study, we evaluated the diagnostic performance of serum *P. jirovecii* PCR and β-D-glucan for PCP diagnosis. Adult patients with hematologic malignancies and positive *P. jirovecii* PCR in lower respiratory tract samples were included as cases and classified as PCP or colonization. Controls had negative BAL PCR. Stored serum samples were analyzed by *P. jirovecii* PCR and β-D-glucan. Forty-one cases (29 PCP, 12 colonization) and 36 controls were included. Serum *P. jirovecii* PCR was positive in 20/41 cases (49%) and in 15/29 patients with PCP (52%), while no controls were serum PCR-positive. For PCP diagnosis, serum PCR had a sensitivity of 52% and a specificity of 89%. For detection of *P. jirovecii* in the lower respiratory tract, sensitivity was 49% and specificity 100%. Detectable serum *P. jirovecii* DNA was associated with higher *P. jirovecii* load in respiratory tract samples and higher β-D-glucan levels, but not with the presence or severity of pneumonia. Serum *P. jirovecii* PCR is a highly specific marker of *P. jirovecii* in the lower respiratory tract and may serve as a useful initial non-invasive test in patients with suspected PCP, prior to invasive respiratory sampling.

## 1. Introduction

*Pneumocystis jirovecii* (Pj) is an opportunistic fungus that causes pneumonia in immunocompromised patients with impaired T-cell immunity. Among patients with haematologic malignancies, patients with acute lymphoblastic leukemia, lymphoproliferative disorders, and hematopoietic stem cell transplant (HSCT) recipients are at particularly high risk [1]. *Pneumocystis* pneumonia (PCP) can lead to acute respiratory failure requiring mechanical ventilation, and mortality rates of up to 50% have been reported [2,3].

Historically, the diagnosis of PCP relied on microscopic detection of Pj with immunofluorescent microscopy (IF) in respiratory tract specimens. However, in recent years, PCR has gained widespread use in the diagnosis of PCP due to its greater sensitivity compared to IF [4,5]. Pj can colonize the lower respiratory tract, and sensitive diagnostic methods such as PCR may also detect the fungus in patients without pneumonia. Considering that the Pj load in the lower respiratory tract is generally higher in patients with pneumonia than in patients who are colonized, the concentration of Pj DNA in the sample, reflected by the PCR Cycle threshold (Ct) value, may help differentiate pneumonia from colonization [6].

In patients with PCP, the concentration of fungus is highest in the distal part of the respiratory tract, i.e., in the alveoli, and lower respiratory tract samples are therefore recommended for sensitive PCP diagnosis [7]. However, PCP is typically characterized by a non-productive cough, and sputum samples may be difficult to obtain. Bronchoscopy to obtain bronchoalveolar lavage fluid (BALF) is therefore often required, exposing patients to potential complications associated with its invasive nature.

In the 1990s, some studies showed that Pj DNA could be detected by conventional PCR in blood samples from HIV patients with PCP, albeit with a low sensitivity [8,9,10,11,12,13]. Over the last few decades, PCR technology has been optimized to yield increased analytical sensitivity. Nevertheless, up-to-date data on the diagnostic performance of Pj PCR in blood remain limited. In a previous study by our group, Pj DNA was detected in serum in 26/26 HIV patients with PCP, yielding a sensitivity of 100% [14]. PCP in patients with HIV infection is typically characterized by a higher Pj burden and a lower degree of inflammation than PCP in patients with other immunosuppressive conditions [15]. Whether Pj DNA can be detected in blood to the same extent in non-HIV patients with PCP, such as patients with haematologic malignancies, remains uncertain.

β-D-glucan (BDG) is a polysaccharide that constitutes a structural component of the cell wall of most pathogenic fungi, including Pj. BDG can be measured quantitatively in serum and has been shown to have a high sensitivity for the diagnosis of PCP [7]. However, BDG is not specific for Pj and may therefore primarily serve as a complement to other diagnostic methods, such as PCR. For example, in patients with detectable Pj DNA in respiratory tract samples, BDG may help distinguish colonization from PCP [16].

The aim of this study was to retrospectively investigate the diagnostic potential of serum-based diagnosis of PCP using BDG and real-time PCR for the detection of Pj DNA in patients with hematologic malignancies. A serum-based diagnostic algorithm could provide a safer, faster, and less resource-intensive alternative to diagnostic procedures that require lower respiratory tract sampling.

## 2. Materials and Methods

We conducted a retrospective case–control study at Sahlgrenska University Hospital in Gothenburg, Sweden. Cases and controls were identified through the Laboratory Information System. Cases who met the following inclusion criteria were included consecutively during the period of 2014 to 2021: (1) ≥18 years of age, (2) a hematological malignancy, (3) a positive Pj PCR result in a lower respiratory tract sample, and (4) availability of a serum sample analyzed for BDG, collected within ±5 days of the respiratory tract sample and stored at −80 °C. Medical records were reviewed to identify any of the two exclusion criteria: absence of radiologic imaging required to distinguish PCP from Pj colonization and initiation of PCP treatment more than one day prior to collection of the serum sample.

Negative controls who met the following inclusion criteria were included consecutively from 2018 until reaching a 1:1 ratio to the cases: (1) ≥18 years of age, (2) a hematological malignancy, (3) a negative Pj PCR result in a BALF sample, (4) availability of a serum sample analyzed for BDG, collected within ±5 days of the respiratory tract sample and stored at −80°C, and (5) no microbiologic evidence of another invasive fungal infection.

Medical records were reviewed to collect background information on cases and controls and to collect data on clinical characteristics among the cases that were considered likely to influence the probability of detecting Pj DNA in serum. Cases with positive Pj PCR in the lower respiratory tract were classified as PCP or Pj colonization according to the criteria of the European Organization for Research and Treatment of Cancer and the Mycoses Study Group (EORTC/MSG) [17,18]. The severity of PCP was assessed based on the degree of hypoxemia, expressed as the PaO_2_/FiO_2_ ratio, on the same day as the serum sample was collected. When arterial blood gas data were unavailable, PaO_2_ was estimated from peripheral oxygen saturation as described elsewhere [19]. PCP severity was categorized as mild-to-moderate if PaO_2_/FiO_2_ was >200 mm Hg (respiratory Sequential Organ Failure Assessment [SOFA] score 0–2) and severe if PaO_2_/FiO_2_ was ≤200 mm Hg (respiratory SOFA score 3–4) [20,21].

BDG had previously been analyzed on the serum samples as part of routine diagnostic laboratory testing. The colorimetric Glucatell assay kit (Associates of Cape Cod, Falmouth, MA, USA) was used according to the manufacturer’s instructions, with a modification including heat pretreatment of samples at 75 °C. The Glucatell assay is the initial BDG assay developed by Associates of Cape Cod, which was later FDA-approved and made available as a commercial diagnostic test under the name of the Fungitell assay. BDG results were reported as concentrations within the measurable range of the assay (50–400 pg/mL). During the latter part of the study period, the method for BDG analysis at the clinical microbiology laboratory was modified to include an extra 1:2 dilution step to yield a more clinically relevant detection range of 100–800 pg/mL.

Lower respiratory tract specimens had previously been analyzed for Pj by PCR as part of routine clinical diagnostics. Subsequently, serum samples from cases and controls stored at −80 °C were thawed and analyzed. Real-time PCR was performed using the BD MAX Open Mode System (BD Diagnostics, Franklin Lakes, NJ, USA), as previously described in detail [14]. In brief, the primers and probe targeting the mitochondrial large subunit ribosomal RNA (mtLSU) gene of Pj, originally described by Dini et al. [22], were used with slight modifications of the probe. The sequences were as follows: forward primer (5′-AAA TAA ATA ATC AGA CTA TGT GCG ATA AGG-3′), reverse primer (5′-GGG AGC TTT AAT TAC TGT TCT GGG-3′), and TaqMan probe (5′-FAM-AGA TAG TCG AAA GGG AAA C-MGB-3′). Cycling conditions consisted of an initial denaturation at 98 °C for 600 s, followed by 46 cycles of 96 °C for 9 s and 62 °C for 30.7 s. For sample preparation, 500 µL of respiratory specimen pretreated with 75 µL of proteinase K (5 mg/mL), or 500 µL of serum was processed using the BD MAX DNA-1 extraction kit. Results were interpreted using a fluorescence threshold of 100 units, together with a valid specimen processing control (SPC). Samples with an invalid SPC were retested after a 1:2 dilution in phosphate-buffered saline. Samples were considered PCR-positive when a distinct amplification curve was obtained. The Pj PCR assay is an established in-house method used for routine diagnostics at the Clinical Microbiology Laboratory. Prior to clinical implementation for respiratory tract specimens, the assay was validated on the BD MAX Open Mode platform by assessing analytical sensitivity, analytical specificity, and reproducibility using positive and negative control samples in accordance with routine laboratory procedures. The PCR analyses were performed by certified laboratory technicians who were blinded to the clinical data of the patients.

Statistical analyses were performed using GraphPad Prism 10.2.2 (GraphPad Software, LLC). Comparison of proportions was performed using Fisher’s exact test, and comparison of medians was performed using the Mann–Whitney U-test. Sensitivity and specificity were calculated by cross-tabulation and are presented with 95% confidence intervals (CI). Analyses were conducted for (A) patients with PCP versus patients without PCP (i.e., patients with Pj colonization and negative controls) and for (B) patients with positive Pj PCR in a lower respiratory tract sample (i.e., patients with PCP and Pj colonization) versus patients with a negative Pj PCR in a lower respiratory tract sample (i.e., negative controls). Estimated predictive values at different assumed pre-test probability scenarios were calculated using Bayes’ theorem, based on the sensitivity and specificity estimates obtained in this study. Correlation between Pj PCR Ct values in respiratory tract and serum samples, as well as between BDG and Pj PCR Ct values in serum, was assessed using Spearman’s correlation coefficient. To account for truncated BDG results and different assay detection limits, concentrations below the limit of detection of 100 pg/mL were set to 99 pg/mL, and concentrations above the limit of detection of 400 pg/mL were set to 401 pg/mL for statistical analysis.

## 3. Results

### 3.1. Characteristics of Cases and Controls

**Figure 1:**
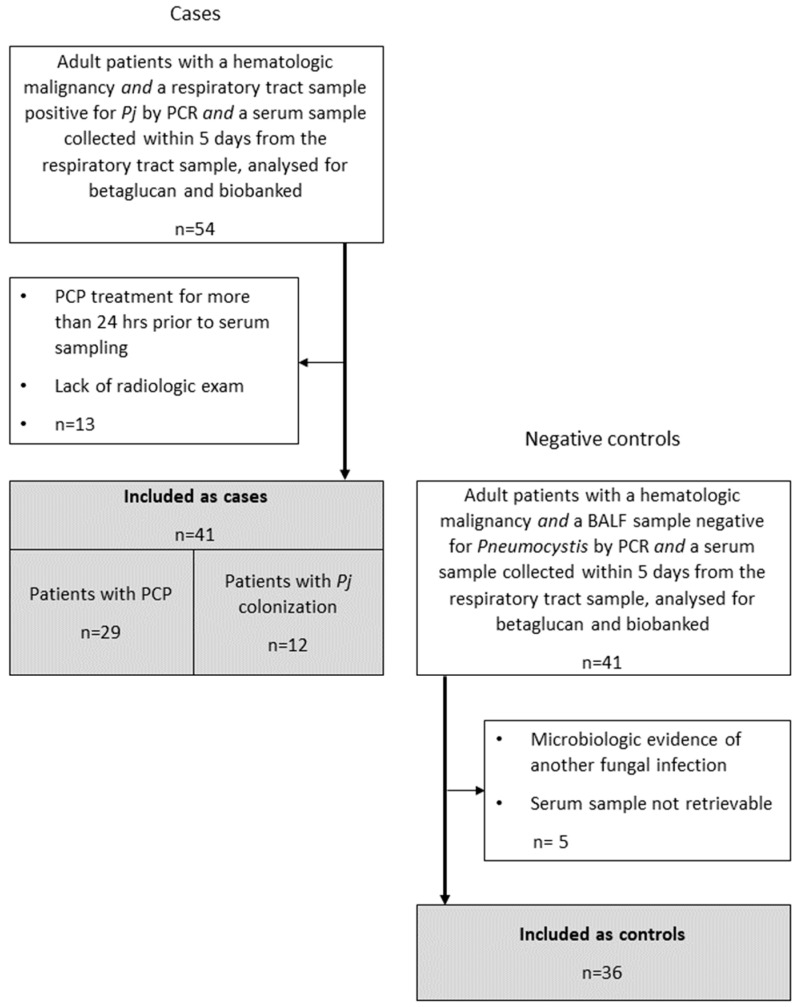
Inclusion of cases and controls.

The inclusion process is shown in Figure 1. In total, 41 patients were included as cases, of whom 29 fulfilled the criteria for PCP and 12 were classified as having Pj colonization. Among 41 initially included controls, 5 were excluded due to non-retrievable serum samples or microbiologic evidence of another fungal infection, leaving 36 patients as negative controls. Baseline characteristics of cases and controls are presented in Table 1. Among the cases, the majority had a lymphoid malignancy (54%), and 37% had undergone HSCT. Twenty patients (49%) had detectable Pj DNA in serum, whereas none of the negative controls had detectable Pj DNA in serum.

### 3.2. Clinical and Microbiological Characteristics According to Serum P. jirovecii PCR Results

Clinical characteristics of the cases, overall and stratified by serum Pj PCR results, are shown in Table 2. Patients with detectable Pj DNA in serum had higher BDG levels and higher Pj load in the lower respiratory tract (i.e., lower PCR Ct values) compared to patients without detectable Pj DNA in serum. In a subgroup analysis of the 29 patients with PCP, there were no significant differences in the severity of pneumonia (assessed by PaO_2_/FiO_2_) or degree of inflammation (defined by the CRP level) between patients with positive and negative serum Pj PCR results.

**Figure 2:**
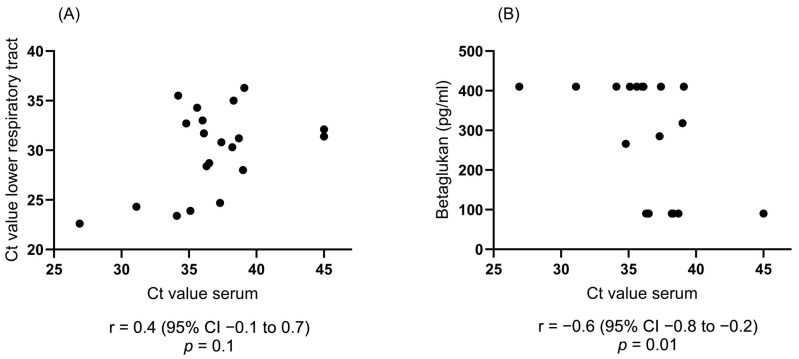
Correlation between Pj PCR Ct values in serum and (**A**) Pj PCR Ct values in the lower respiratory tract, and (**B**) BDG levels in serum in patients with detectable serum Pj DNA. Correlation was assessed by Spearman’s correlation coefficient and was based on 20 patients with a positive Pj PCR in serum (15 patients with PCP and 5 patients with Pj colonization). For the analysis of BDG, one patient with a recent invasive candidiasis was excluded.

In patients with positive Pj PCR results in serum, there was a moderate to strong negative correlation between Pj PCR Ct values in serum and BDG levels (r = −0.6, *p* = 0.01, Figure 2). There was a trend towards a moderate positive correlation between Pj PCR Ct values in serum and Pj PCR Ct values in the lower respiratory tract (r = 0.4), although this did not reach statistical significance.

### 3.3. Diagnostic Performance of Pj PCR and BDG in Serum

The diagnostic performance of serum analyses (BDG and Pj PCR) for diagnosing PCP and for identifying patients with the presence of Pj in the lower respiratory tract is summarized in Table 3. Estimated positive and negative predictive values of serum analyses to diagnose PCP and to identify the presence or absence of Pj in the lower respiratory tract at different assumed pre-test probabilities scenarios are shown in Table 4. Serum Pj PCR had a positive predictive value (PPV) of 100% to correctly identify patients with the presence of Pj in the lower respiratory tract, regardless of pre-test probability. For PCP diagnosis, serum Pj PCR had a PPV of 20% at a pre-test probability of 5% and 83% at a pre-test probability of 50%.

## 4. Discussion

In this study, we investigated the diagnostic potential of serum-based analyses for PCP diagnosis in patients with hematologic malignancies. Serum Pj PCR showed moderate sensitivity but excellent specificity for detecting Pj in the lower respiratory tract, supporting its potential role as an initial non-invasive test prior to invasive respiratory sampling. Detectable Pj DNA in serum was associated with a higher Pj load in respiratory tract samples, but not with the presence or severity of *Pneumocystis* pneumonia.

Previous studies evaluating serum-based PCR for the diagnosis of PCP have mainly been conducted in patients with HIV infection [23]. Many of these studies relied on conventional PCR techniques with lower analytical sensitivity [8,9,10,11,12,13,24], which limit their applicability to current clinical practice. In addition, these studies include PCR methods with different gene targets, which further complicates direct comparisons between results. However, in a more recent study by our group, where we used real-time PCR targeting the mitochondrial large subunit (mtLSU) rRNA gene, 100% of HIV patients with a diagnosis of PCP had detectable Pj DNA in serum [14]. Two other studies on HIV patients, using real-time PCR targeting the same gene, reported substantially lower sensitivities. One study reported a sensitivity of 57% [25], but this study included patients with probable PCP who had received up to two days of treatment. Another study reported detectable Pj DNA in serum in only 25% of HIV patients with a clinical picture compatible with PCP [26]. In that study, no patients had received prior treatment, but the case definition of PCP was not clearly defined, and microbiologic data on Pj in the respiratory tract were lacking, making the results difficult to interpret. In a meta-analysis on non-invasive diagnosis of PCP, the authors conclude that data on diagnostic performance of Pj PCR analyzed in serum are limited and that further studies on non-HIV immunocompromised patients are warranted [23].

In our study, which exclusively included patients with hematologic malignancies, 49% of patients with Pj detected in the lower respiratory tract—i.e., patients with PCP or colonization—had detectable Pj DNA in serum. Similar results were observed in the subgroup of patients classified as PCP, where the sensitivity of serum Pj PCR was 52%. Because PCP in non-HIV immunocompromised patients represents a distinct clinical and microbiological entity from HIV-associated PCP [15], the diagnostic performance of Pj PCR in blood observed in patients with HIV may not be directly applicable to non-HIV immunocompromised populations. Only a few previous studies have investigated the diagnostic performance of real-time Pj PCR on blood samples in patients with non-HIV-related PCP. One study, including 30 patients with various non-HIV immunosuppressive conditions, reported an overall sensitivity of 25% [27]. In another study, in which 77% of patients with PCP had an underlying immunosuppressive condition other than HIV, circulating cell-free Pj DNA in plasma was detected in 49% of cases [28]. Despite incorporating different methodologies for DNA detection, these results are consistent with our results and indicate that the diagnostic performance of Pj PCR in blood may be lower in patients with non-HIV-related PCP. When BDG was incorporated into the serum-based diagnostic algorithm in our study, the sensitivity did not improve. In total, 46% of patients with PCP and 41% of patients with Pj detected in the lower respiratory tract had BDG levels above the defined cut-off of 200 pg/mL, indicating that the serum Pj PCR assay outperformed the BDG assay in this setting.

Factors influencing the likelihood of detecting Pj DNA in blood are not fully understood. In our study, there was no correlation between the severity of pneumonia (measured by the PaO_2_/FiO_2_ ratio) or the degree of inflammation (measured by CRP-level) and the detection rate of Pj DNA in serum. On the other hand, the Pj load in the respiratory tract was higher, reflected by lower Pj PCR Ct values, in patients with detectable Pj DNA in serum. Similar observations were reported in the aforementioned studies of non-HIV PCP. Costa et al. found that 90% of patients with a higher Pj burden in BAL—defined as Pj detected by immunofluorescence microscopy—had detectable Pj DNA in blood, compared with 0% among patients with a lower Pj burden (i.e., PCR-positive but microscopy-negative BAL samples) [27]. Likewise, Moreno et al. reported a sensitivity of 100% for circulating cell-free Pj DNA in plasma among patients with microscopy-positive PCP, but only 30% among patients with PCR-positive PCP [28]. Taken together, these observations indicate that it is the Pj load, rather than the underlying immunosuppressive condition or the severity of pneumonia, that predicts serum Pj PCR positivity. The higher Pj burden typically observed in HIV-associated PCP compared with PCP in patients with other immunosuppressive conditions [15] may therefore explain the higher sensitivity of Pj PCR in serum observed in our previous study of patients with HIV [14]. We also found that BDG levels were higher in patients with detectable Pj DNA in serum compared with those without. This association likely reflects higher Pj loads in the respiratory tract, consistent with previous studies reporting a correlation between *Pneumocystis* fungal burden and serum BDG levels [16,27,29].

The specificity of serum Pj PCR was 100% when assessed against controls with negative Pj PCR in the lower respiratory tract, whereas the specificity of BDG was 92% (at a cut-off of 200 pg/mL). Similarly, previous studies in predominantly non-HIV immunosuppressed patients reported specificities of >99% for serum Pj PCR when controls were negative for Pj in the respiratory tract [27,28]. In line with our findings, Moreno et al. also observed lower specificity for BDG (67% at a cut-off of 80 pg/mL) than for cell-free Pj DNA in blood [28].

In a clinical context, predictive values are more informative for decision-making than sensitivity and specificity, as they account for the patient’s pre-test probability across different clinical scenarios. Accordingly, we analyzed the predictive values of Pj PCR and BDG in serum across different assumed pre-test probability scenarios, assessing both the probability of having PCP and the probability of having *Pneumocystis* in the lower respiratory tract. In a screening scenario for a typical risk group, a 5% pre-test probability of PCP could be expected, similar to the 6% cumulative incidence observed in patients with Hodgkin’s lymphoma [30]. At this pre-test probability, a positive Pj PCR result in serum corresponded to a 20% probability of having PCP, and a positive Pj PCR combined with BDG >200 pg/mL increased this probability to 32%. These findings indicate that, in settings with low pre-test probability, these tests are not sufficiently accurate to rule in PCP. Conversely, at a pre-test probability of 25%, reflecting a situation with a high clinical suspicion of PCP, the positive predictive value of Pj PCR alone was 61%, increasing to 75% when combined with a BDG >200 pg/mL. Thus, while neither test is reliable for confirming PCP in low-risk scenarios, they may be useful in high-risk scenarios, particularly when used together.

Analysis of predictive values for the probability of having Pj in the lower respiratory tract demonstrated that Pj PCR in serum had a 100% positive predictive value, even at a low pre-test probability of 5%, whereas BDG showed substantially lower predictive values. This suggests that a positive Pj PCR in serum is highly indicative of *Pneumocystis* colonization or PCP and may serve as a surrogate marker for Pj DNA in the lower respiratory tract.

Our study has several limitations. The retrospective case–control design may introduce selection bias and can overestimate diagnostic accuracy compared with prospective cohort studies, since cases and controls are selected rather than sampled from a general at-risk population. To mitigate this, we included consecutive negative controls from the same risk population who underwent respiratory tract sampling under the same clinical conditions and for the same indications as the cases, creating a more comparable and clinically relevant sample. We also analyzed predictive values across a range of hypothetical pre-test probabilities, allowing interpretation in the context of different clinical scenarios and better reflecting real-world diagnostic performance. Additionally, all patients with Pj in the lower respiratory tract, as well as those with colonization, were included to more accurately represent real-world conditions. Nevertheless, the relatively small number of patients reduces the precision of our estimates and may limit generalizability. Despite these limitations, our findings provide valuable insights into the predictive value of Pj PCR and BDG in serum and their potential role in clinical decision-making, highlighting areas for future prospective studies with larger cohorts.

## 5. Conclusions

In conclusion, our study demonstrates that serum Pj PCR is a highly specific marker for the presence of *Pneumocystis* in the lower respiratory tract, with positivity reflecting higher airway Pj load, whereas BDG is less specific. Although serum Pj PCR cannot reliably distinguish PCP from colonization, it may serve as a useful initial diagnostic tool, since a positive result could preclude the need for lower respiratory tract sampling in patients with compatible risk factors and clinical features compatible with PCP. These findings support the incorporation of serum-based Pj PCR into diagnostic algorithms, although prospective studies in larger cohorts are needed to further define its clinical utility across different immunocompromised populations.

## Figures and Tables

**Table 1:** Baseline characteristics of cases and controls.

		Cases			Controls
		All	PCP	Pj Colonization	
		*n* = 41	*n* = 29	*n* = 12	*n* = 36
Age, yrs, median (range)		66 (23–78)	67 (23–78)	65 (25–73)	54 (20–74)
Sex, *n* (%)					
	Male	25 (61)	19 (66)	6 (50)	19 (53)
Hematologic diagnosis					
Lymphoid malignancy, *n* (%)		22 (54)	17 (59)	5 (42)	8 (22)
	B-cell lymphoma, *n* (%)	12 (29)	9 (31)	3 (25)	3 (8)
	T-cell lymphoma, *n* (%)	3 (7)	3 (10)	0 (0)	0 (0)
	ALL, *n* (%)	1 (2)	0 (0)	1 (8)	5 (14)
	CLL, *n* (%)	6 (15)	5 (17)	1 (8)	0 (0)
Myeloid malignancy, *n* (%)		9 (22)	5 (17)	4 (33)	25 (69)
	AML/MDS, *n* (%)	9 (22)	5 (17)	4 (33)	23 (64)
	CML, *n* (%)	0 (0)	0 (0)	0 (0)	2 (6)
Plasma cell malignancy, *n* (%)		6 (15)	6 (21)	0 (0)	1 (3)
	Myeloma, *n* (%)	5 (12)	5 (17)	0 (0)	1 (3)
	Mb. Waldenström, *n* (%)	1 (2)	1 (3)	0 (0)	0 (0)
Other ^a^, *n* (%)		4 (10)	1 (3)	3 (25)	2 (6)
HSCT, *n* (%)		15 (37)	7 (24)	8 (67)	16 (44)
	Allogenic, *n* (%)	10 (24)	3 (10)	7 (58)	14 (39)
	Autologous, *n* (%)	5 (12)	4 (14)	1 (8)	3 (6)
Detectable Pj DNA in serum, *n* (%)		20 (49)	15 (52)	5 (42)	0 (0)

ALL: acute lymphoblastic leukemia. CLL: chronic lymphocytic leukemia. AML: acute myeloid leukemia. MDS: myelodysplastic syndrome. CML: chronic myeloid leukemia. HSCT: hematopoietic stem cell transplantation. ^a^ Aplastic anemia, myelofibrosis.

**Table 2:** Clinical characteristics of the cases, overall and stratified by the result of serum Pj PCR.

			Overall	Stratified by Result of Pj PCR in Serum		
				Positive	Negative	*p*
All cases			*n* = 41	*n* = 20	*n* = 21	
Age, yrs, median (range)			66 (23–78)	65 (23–77)	69 (32–78)	ns
PCP, *n* (%)			29 (71)	15 (75)	14 (67)	ns
Death at 30 days, *n* (%)			3 (7)	2 (10)	1 (5)	ns
BDG level, median (range)			99 (99–401) ^a,b^	318 (99–401) ^a^	99 (99–401) ^b^	0.01
BDG level > 200 pg/mL, *n* (%)			16 (41) ^a,b^	12 (63) ^a^	4 (20) ^b^	0.01
Ct value airway specimen, median (range)			32 (23–38)	31 (23–36)	33 (27–38)	0.03
Patients with PCP			*n* = 29	*n* = 15	*n* = 14	
	BDG level, median (range)		125 (99–401) ^a^	360 (99–401) ^a^	99 (99–401)	0.02
	BDG level > 200 pg/mL, *n* (%)		13 (46) ^a^	10 (71) ^a^	3 (21)	0.02
	Ct value airway specimen, median (range)		32 (23–38)	29 (23–36)	32 (27–38)	ns
	Duration of symptoms, median days (range)		12 (1–43)	16 (1–43)	12 (6–37)	ns
	CRP, median (range)		110 (11–370)	64 (11–370)	130 (16–310)	ns
	PAO_2_/FiO_2_, median (range)		376 (151–457)	376 (151–457)	362 (153–457)	ns
		SOFA mild (0–2), *n* (%)	25 (86)	13 (87)	12 (86)	ns
		SOFA severe (3–4), *n* (%)	4 (14)	2 (13)	2 (14)	ns
	Death at 30 days, *n* (%)		3 (10)	2 (13)	1 (7)	ns
Patients with Pj colonization			*n* = 12	*n* = 5	*n* = 7	
	BDG level, median (range)		99 (99–401) ^b^	99 (99–401)	99 (99–401) ^b^	-
	BDG level > 200 pg/mL, *n* (%)		3 (27) ^b^	2 (67)	1 (17) ^b^	-
	Ct value airway specimen, median (range)		35 (29–38)	31 (30–36)	35 (29–38)	-

ns: not statistically significant. Owing to the small number of patients with Pj colonization, no statistical analyses were performed in this subgroup. ^a^ one patient with PCP and recent invasive candidiasis was excluded due to the risk of biased BDG result. ^b^ one patient with Pj colonization and recent invasive aspergillosis was excluded due to the risk of biased beta-glucan result.

**Table 3:** Diagnostic performance of serum analyses (**A**) to diagnose PCP and (**B**) to identify patients with presence of Pj in the lower respiratory tract.

**(A) To Diagnose PCP**		
	**Sensitivity ^a^, % (95% CI)**	**Specificity ^b^, % (95% CI)**
Pj PCR	52 (34–69)	89 (78–95) ^c^
BDG ^d^	46 (30–64) ^e^	87 (75–94) ^f^
Pj PCR and BDG ^d^	36 (21–54) ^e^	96 (86–99) ^c,f^
**(B) To identify the presence of Pj in the lower respiratory tract**		
	**Sensitivity ^g^, % (95% CI)**	**Specificity ^h^, % (95% CI)**
Pj PCR	49 (34–63)	100 (90–100) ^c^
BDG ^d^	41 (27–57) ^e,f^	92 (78–97)
Pj PCR and BDG ^d^	31 (19–46) ^e,f^	100 (90–100) ^c^

^a^ analysis based on 29 patients with PCP. ^b^ analysis based on 48 patients without PCP (patients with Pj colonization and negative controls). ^c^ one negative control excluded due to indeterminate PCR-result in serum. ^d^ cut-off 200 pg/mL. ^e^ one patient with PCP excluded due to recent invasive candidiasis. ^f^ one patient with Pj colonization excluded due to recent invasive aspergillosis. ^g^ analysis based on 41 patients with positive Pj PCR in lower respiratory tract sample (PCP and Pj colonization). ^h^ analysis based on 36 negative controls.

**Table 4:** Estimated predictive values of serum analyses (**A**) to diagnose PCP and (**B**) to identify patients with presence of Pj in the lower respiratory tract at different assumed pre-test probabilities scenarios.

**(A) To Diagnose PCP**						
	**Positive Predictive Value, %**			**Negative Predictive Value, %**		
	Pj PCR	BDG ^a^	Pj PCR and BDG ^a^	Pj PCR	BDG ^a^	Pj PCR and BDG ^a^
Pre-test probability						
5%	20	16	32	97	97	97
15%	45	38	61	91	90	89
25%	61	54	75	85	83	82
50%	83	78	90	65	62	60
**(B) To identify the presence of Pj in the lower respiratory tract**						
	**Positive predictive value, %**			**Negative predictive value, %**		
	Pj PCR	BDG ^a^	Pj PCR and BDG ^a^	Pj PCR	BDG ^a^	Pj PCR and BDG ^a^
Pre-test probability						
5%	100	21	100	97	97	96
15%	100	47	100	92	90	89
25%	100	63	100	85	82	81
50%	100	84	100	66	61	59

Calculations at different assumed pre-test probabilities scenarios were done using Bayes’ theorem based on the sensitivity and specificity presented in Table 3 and with the groups “Patients with PCP or Pj colonization” vs. “negative controls”. ^a^ cut-off 200 ρg/mL.

## Data Availability

The raw data supporting the conclusions of this article will be made available by the authors on request.

## Abbreviations

The following abbreviations are used in this manuscript:

PCR	Polymerase chain reaction
PCP	*Pneumocystis jirovecii* pneumonia
BDG	β-D-glucan
Pj	*Pneumocystis jirovecii*
